# Traumatic tricuspid valve papillary muscle case with concomitant acquired patent foramen ovale and covert right atrial rupture

**DOI:** 10.5935/0103-507X.20190034

**Published:** 2019

**Authors:** Evangellos Pertsas, Theodoros Aslanidis, Georgios Andricopoulos, Vasilios Gulielmos

**Affiliations:** 1 Intensive Care Unit, St. Paul General Hospital - Thessaloniki, Greece.; 2 Department of Cardiac Surgery, "Geniki Kliniki" Clinic - Thessaloniki, Greece.

**Keywords:** Tricuspid valve/injuries, Echocardiography, Valva tricúspide/lesões, Ecocardiografia

## Abstract

Cardiac trauma often occurs in motor vehicle accidents. A 50-year-old female driver was transported to our hospital with multiple trauma after a high-speed car accident. After admission to the intensive care unit, cardiac ultrasound examination revealed traumatic tricuspid valve papillary muscle rupture and patent foramen ovale, while Lancisi's sign was noted on physical examination. Surgical treatment was performed with valve annuloplasty and closure of the patent foramen ovale and a covert right atrial defect that was detected intraoperatively.

## Abstract (pt)

O traumatismo cardíaco é comum em acidentes com veículos automotores. Uma mulher com 50 anos de idade foi transportada para nosso hospital após sofrer múltiplos traumatismos em um acidente de automóvel quando dirigia em alta velocidade. Após admissão à unidade de terapia intensiva, uma ultrassonografia cardíaca revelou ruptura traumática de músculo papilar da valva tricúspide e forame oval patente, enquanto se observou, no exame físico, o sinal de Lancisi. Foi realizado tratamento cirúrgico com anuloplastia da valva e fechamento do forame oval patente; durante o ato cirúrgico, diagnosticou-se ruptura oculta do átrio direito.

## INTRODUCTION

Cardiac trauma occurs mostly due to motor vehicle accidents. Patients vary widely in the severity of their condition on presentation, and related mortality remains high despite improvements in diagnosis and management.^(1)^

The present article presents a case of traumatic tricuspid insufficiency due to tricuspid valve papillary muscle rupture with a concomitant right-to-left atrial shunt across a patent foramen ovale (PFO).

The draft was prepared in accordance with the CARE guidelines.^(2)^

## CASE REPORT

A 50-year-old Caucasian female driver was brought to the Emergency Department following a high-speed car accident. Vehicle extrication had to be performed on the scene (duration 35 minutes). No information about seat belt use was available. Her medical history included arterial hypertension and depression. Her drug regimen included nebivolol 5mg d.i.d., lamotrigine 25mg b.i.d. and fluoxetine 25mg o.d. No allergies were mentioned.

On admission, she presented with a Glasgow Coma Scale of E2/V3/M5 (Eye/Verbal/Motor response), heart rate (HR) 70 beats/min, blood pressure 65/37mmHg, core temperature (t°) 34°C, respiratory rate 9 breaths/min, oxygen pulse saturation (SpO_2_) 85% on oxygen mask (flow - 15L/min), mixed lung sounds on both sides upon auscultation, and bruises all over the right upper limb and both lower limbs. Moreover, right leg length discrepancy with concomitant right knee outer rotation was noted. Alcohol odor on breath was recorded. Full spine immobilization and 1.2L of crystalloids had already been given by the Emergency Medical Technicians. Her Revised Trauma Score was 4 and Emergency Trauma Score was 7.

Rapid sequence intubation was performed, and further investigation (computed tomography (CT), CT angiography and X-ray imaging) revealed multiple rib fractures on both sides (4^th^ - 6^th^ ribs on the left and 9^th^ - 12^th^ ribs on the right), sternal body fracture, small left pneumothorax, left lung contusions, right hepatic lobe contusion, and right distal femoral fracture with knee involvement.

**Figure 1:**
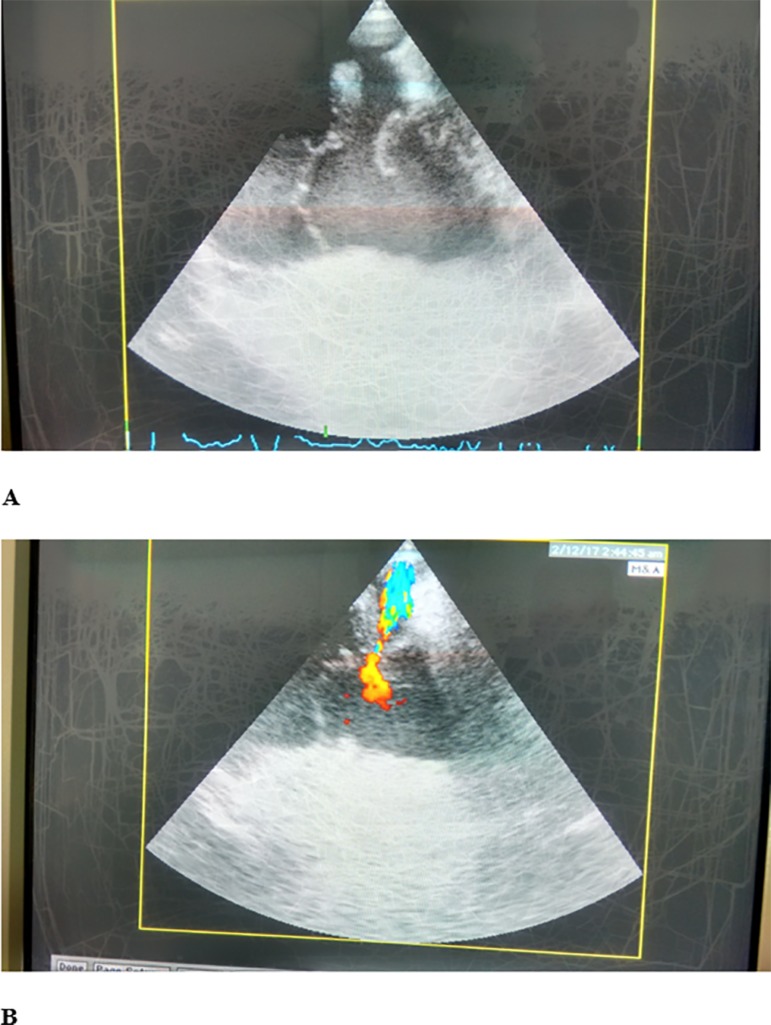
Snapshots during transesophageal echocardiography examination (transverse plane, long axis view) revealing traumatic tricuspid valve regurgitation.

**Figure 2:**
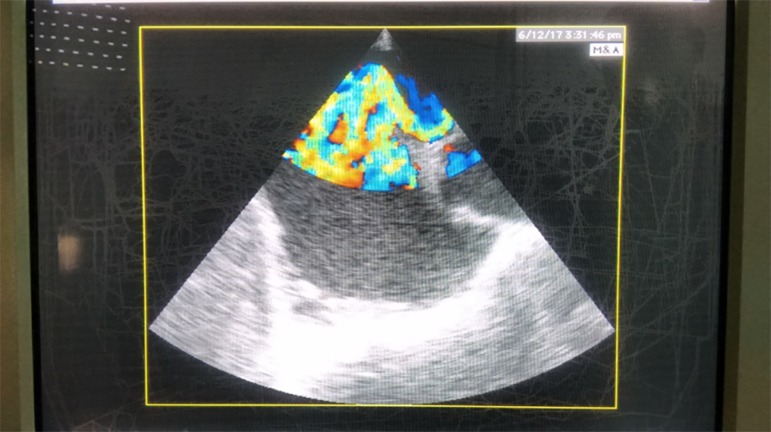
Snapshot during second transesophageal echocardiography examination (transverse plane, basal short axis) revealing patent foramen ovale.

On admission to the intensive care unit (ICU), the patients Acute Physiology and Chronic Health Evaluation (APACHE) II score was 31, and her *Sequential Organ Failure Assessment* (SOFA) score was 11. An arterial blood gas exam revealed severe mixed acidosis: pH 7.22, partial pressure of oxygen 54.2mmHg, partial pressure of carbon dioxide 37.9mmHg, potassium 3.5mEq/L, sodium 140mEq/L, bicarbonate15mEqL, basis excess 11.8, anion gap (corrected for albumin) 13.25mmol/L, and lactate 10.4mmol/L. Further laboratory results were hemoglobin 7.8g/dL, leukocytes 13.4k/dL, platelets 260k/dL, partial prothromboplastin time 32 sec, prothrombin time 19.2 sec, serum creatinine 1.22mg/dL, urea 34mg/dL, serum glutamic oxaloacetic transaminase 342IU/L, serum glutamic-pyruvic transaminase (SGPT) 221IU/L, creatine phosphokinase 2716IU/L, and troponin T 327mcg/L. Central venous pressure measurement was 7cmH_2_O, and bulging of the right jugular vein was noted (Lancisi's sign). A grade 2/6 holosystolic murmur that was augmented during inspiration was noted at the left lower sternal border. Transthoracic echocardiography (TTE) revealed signs of possible tricuspid valve injury. Subsequent transesophageal echocardiography (TEE) revealed traumatic tricuspid valve papillary muscle injury (Figures 1A and B). During the following hours, positive end-expiratory pressure (PEEP)-dependent (from 7 to 4cmH_2_O) hypoxia was noted, and so a second TEE was performed. Injection of a micro-bubble through the central line revealed a PFO, which was also displayed with color Doppler (Figure 2). Right ventricular systolic pressure was 42 - 44mmHg, yet with preserved systolic function. At the time, and for the rest of her ICU hospitalization, plateau pressure was < 25mmHg and tidal volume was < 8mL/kg.

Stabilization of the patient continued with fluid resuscitation and high doses of vasopressors (noradrenaline 1.1µg/kg/min), active rewarming and reversal of acid-base derangement.

Due to lack of a cardiosurgical unit in the hospital, communication with other hospitals in the region was conducted. Transportation of the patient to a private cardiosurgical unit was performed 5 days later.

At operation, a median sternotomy was performed and cardiopulmonary bypass was instituted. The tricuspid valve was exposed through right atriotomy. Intraoperatively, apart from the tricuspid valve papillary muscle rupture and PFO (1cm), a 3cm length covert rupture of the right atrium was also noticed. Correction included tricuspid valve annuloplasty with a Carpentier-Edward ring 28mm and complementing valve leaflets, closure of the PFO and additional atrial wall defect with continuous surgical suture. The postoperative course of the patient was uneventful, and she was discharged from the hospital with a Glasgow Outcome Scale of 7. A year later, the woman had fully recovered, apart from a residual functional defect of the right knee.

## DISCUSSION

Traumatic tricuspid insufficiency (TTI) is a relatively rare condition. Nevertheless, an increasing frequency of such cases has been published during the last 2 decades, mainly due to the wider use of better diagnostic procedures and awareness of this pathology.^(3)^ In addition, because most people tolerate isolated TTI well, the true incidence of the condition is probably underestimated.^(4)^

Tricuspid valve damage may involve leaflets, chordae tendineae or papillary muscles. Isolated TTI is considered rare.^(5)^ Available literature displays a great variety of injuries accompanying TTI: TTI with PFO, TTI, PFO and atrial or ventricular septal defect, TTI and mitral valve rupture, etc.^(5,6)^ In the latter cases, early diagnosis and reduced interval from admission to operation are key points of management.

In our patient, disrupted valve motion due to papillary muscle rupture resulted in an abrupt rise in intra-atrial pressure that led to a right-to-left shunt across the foramen ovale. In the literature, high PEEP (> 9cmH_2_O), plateau pressure > 26mmHg and right ventricle/left ventricle area ratio > 1 have been reported as risk factors for PFO.^(7)^ Thus, mechanical ventilation may be another contributing factor for PFO, although of less importance. Finally, we hypothesize that the atrial wall defect, which was detected intraoperatively and recorded as "covert", did not contribute substantially to the shunt. Transesophagial echocardiography provides a safe and easy method for visualization of the heart, mediastinum and most of the thoracic aorta. Although TTE examination can also provide information about possible structural cardiac damage, TEE is the method of choice for diagnosis and follow-up for acute clinical deterioration.

Unfortunately, until now (2018), the experience with TTI and its accompanying injuries is based on case reports or small case series. A larger trial or a systematic review will give more information about the management strategy of these conditions.

## CONCLUSION

Traumatic tricuspid insufficiency is a condition that needs a high level of awareness in order to detect it early. Concomitant damage to other cardiac structures seems to be the main determinant of the management in each case.
